# Quantifying phenotypic and genetic variation for cow fertility phenotypes in American Simmental using total herd reporting data

**DOI:** 10.1093/jas/skae364

**Published:** 2024-12-19

**Authors:** Cassidy C Catrett, Sarah E Moorey, Jon E Beever, Troy N Rowan

**Affiliations:** Department of Animal Science, University of Tennessee, Knoxville, TN 37996, USA; Department of Animal Science, University of Tennessee, Knoxville, TN 37996, USA; Department of Animal Science, University of Tennessee, Knoxville, TN 37996, USA; Department of Animal Science, University of Tennessee, Knoxville, TN 37996, USA

**Keywords:** cow fertility, genetic selection, heritability, novel phenotypes

## Abstract

Reproduction plays a major role in the production efficiency of livestock species. However, cow-centric reproductive traits tend to be lowly heritable and are not expressed until later in an animal’s lifetime, making phenotypic selection alone inefficient at generating genetic gain. Genetic progress can be accelerated by focusing selection on the predicted genetic component of reproductive traits using Expected Progeny Differences. We used the American Simmental Association’s performance and Total Herd Enrollment data, made up of 533,155 calving records from 303,158 females (132,403 cows and 170,755 heifers), 33,732 of which are genotyped, to explore three continuous and two discrete phenotypes focused on quantifying early and sustained fertility in beef cows. We analyzed calving date (**CD**) (cow’s CD relative to the start of the calving season), calving interval (**CI**) (days between calves), first calving interval (**FCI**) (CI observation between the first and second calving record for a female), heifer pregnancy (**HP**) (did the animal calve as 2-yr-old), and discrete early calving (**DEC**) (did animal calve in the first 30 d of the calving season) as distinct, but correlated measures of fertility. This dataset provides insight into population-wide trends related to cow attrition, calving season lengths, and phenotypic variation in fertility. We used pedigree and genomic REML to estimate these six phenotypes’ genetic, permanent environment, and temporary environmental variance components. Pedigree-estimated heritabilities were 0.06 (± 0.000011) for CD, (0.04 ± 0.000005) for CI, 0.07 (± 0.000016) for DEC, 0.05 ± 0.000041 for FCI, and 0.23 (± 0.000099) for HP, consistent with other fertility traits across beef and dairy cattle. The incorporation of genomics increased the heritability estimate for HP (0.24 ± 0.000098) and decreased the estimate for FCI (0.04 ± 0.000029). Positive phenotypic and genetic correlations were found among these phenotypes (r_G_** **= 0.01 to 0.96). These results call for further work in optimizing genetic predictions and exploration of the genetic architecture through genome-wide studies. Whole herd reporting date frameworks represent opportunities for measuring new reproductive phenotypes, but their utility in genetic evaluations will rely on novel trait initiatives and consistent recording that captures more detailed data.

## Teaser

Complete calving records from a large, publicly collected dataset enable the in-depth exploration of beef cattle fertility phenotypes. These findings provide insight into macro-level management trends and pathways forward for genetic improvement.

## Introduction

Reproduction plays a major role in the production efficiency of livestock species. For beef cow-calf operations, calves serve as the main end product and generate the majority of herds’ economic output (Diskin and Kenny, 2014). The ultimate goal for cow productivity is for females to have their first calf by 24 mo of age and then produce a calf every 365 d until they are removed from production. Cows that fail to reproduce or are removed from the herd prior to their breakeven point fail to reach lifetime profitability (Mathews and Short, 2001). This breakeven point is when a female has successfully produced enough calf weight to cover the costs of heifer development and annual maintenance. Boyer et al. (2020) found that this breakeven point is typically after a female has successfully produced six calves by 7 yr of age. However, this largely depends on variable cow costs.

Cow longevity is the chief driver of economic success in cow-calf operations (Garcia et al., 2015). An inability to rebreed is the most common reason that beef cows are prematurely culled from herds (Diskin and Kenny, 2014). Phenotypes associated with increased cow fertility and longevity are known to be lowly heritable (Cammack et al., 2009). As such, selection tools can help accelerate progress on cow fertility by focusing selection on only the modest genetic variation that controls these traits. Measuring these phenotypes at the population level, necessary for genetic evaluations, is challenging with conventional data recording schemes (MacNeil et al., 2006). Conventional reporting schemes do not require female annual cow inventories or performance records for nonregistered animals. Without calving information on all cows, we cannot be certain of reproductive success or failure. This makes traditional datasets unusable for developing fertility phenotypes and creates biases in contemporary group estimates. Further, many of these fertility phenotypes take multiple years to be fully expressed, making phenotypic selection ineffective at generating rapid genetic improvement.

Many measures have been proposed for quantifying heifer and cow fertility. These include calving date (**CD**), age at first calving, heifer pregnancy (**HP**), and CI (Lesmeister et al., 1973; Bourdon and Brinks, 1982; Cundiff et al., 1986; Doyle et al., 2000). However, a lack of comprehensive breeding records in the beef industry has made the collection of these phenotypes difficult. Whole herd reporting (**WHR**) is an inventory-based reporting system where all female production records and calving information are recorded, regardless of calf survival or registration (Giess et al., 2021). This type of reporting framework has allowed breed associations to collect complete and unbiased reproductive and performance phenotypes from entire herds. However, these reporting schemes have not been universally adopted across breed associations, and many reporting schemes lack the granular details necessary for calculating date-dependent reproductive phenotypes (Giess et al. 2021).

Producers have the ability to make selection decisions using genetic predictions in the form of Expected Progeny Differences (**EPDs**). EPDs are statistical estimates of an animal’s additive genetic merit for a given trait. Numerous traits exist that allow producers to make more informed bull selection decisions that will result in more productive replacement females (Rowan, 2022). The two most commonly reported EPDs for female productivity in current genetic evaluations are HP and Stayability (STAY). HP predicts the difference in the percentage of daughters of individuals who will calve as 2-yr-olds (BIF Guidelines, 2023). Stayability is a comprehensive measure of cow longevity that predicts the proportion of an animal’s daughters that will remain in a herd to at least 6 yr of age without missing a calf (Snelling et al., 1995; BIF Guidelines, 2023). While not explicit predictions of sustained fertility, the two traits are strongly linked due to infertility being a major driver of culling. Limited whole herd reporting throughout beef cattle breeds means that these EPDs may be less accurate or noisier than predictions of other traits with more easily collected phenotypes (e.g., weaning weight, birth weight).

Reproductive traits are lowly heritable (typically *h*^2^ < 0.1), and historical improvements have largely relied on changes to management practices rather than genetic improvements (Gutiérrez et al., 2002; Cammack et al., 2009; Minick Bormann and Wilson, 2010; Berry et al., 2014; Boldt et al., 2018). This, coupled with delayed expression and limited information collected on behalf of sires, presents a need for the collection of other economically important reproductive phenotypes. This project aimed to estimate the heritabilities and genetic correlations for multiple cow fertility traits. We used the American Simmental Association’s (**ASA**) Total Herd Enrollment (**THE**) dataset to calculate five phenotypes associated with HP and sustained cow fertility. We hypothesized that cow and heifer fertility and rebreeding phenotypes would be highly variable across the ASA population and under low levels of genetic control. Our major objectives aimed to 1) develop informative reproductive phenotypes using enrollment data and CDs, 2) understand the phenotypic distributions of reproductive phenotypes, particularly throughout a cow’s lifetime, and 3) estimate the role that genetics plays in the expression of these traits. This work sets the stage for further exploration of these reproductive phenotypes, their economic value, genetic evaluation, and genetic architecture.

## Materials and Methods

### Dataset

**Figure 1.**
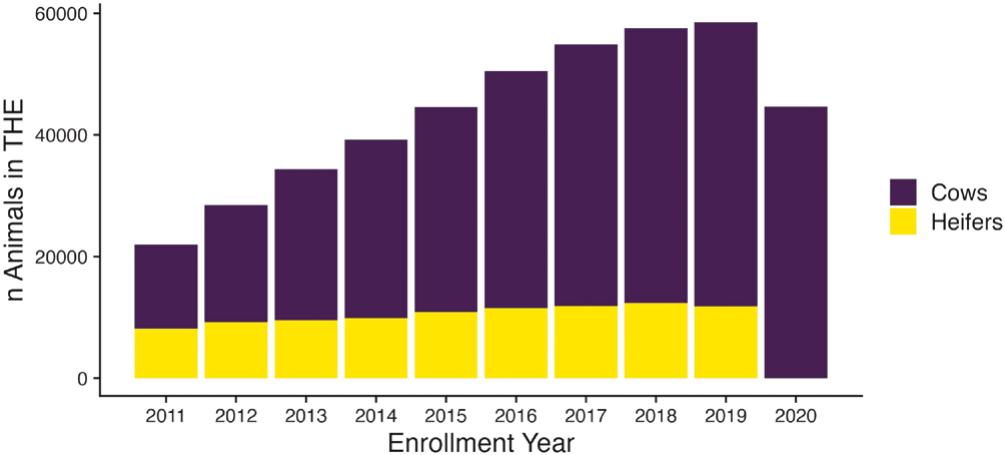
THE records by year: A histogram of the number of enrollment records in THE dataset from the years 2011 to 2020. Bars represent the total number of animals enrolled in THE per year.  The number of cows and heifers enrolled each year are represented by the top and bottom portions of bars, respectively.

As all of the data used in this work was producer-reported in an existing database, no IACUC protocol was necessary. Producer-collected enrollment records originated from the ASA THE program prior to January 1, 2021. Enrollment records for each animal consisted of enrollment year, season, breeder, and productivity and status codes (Giess et al., 2021). The ASA Herdbook is open, meaning that not all animals are purebred Simmentals, and a wide range of breed compositions are present within. All animals had pedigree-estimated breed compositions. We excluded all animals with >1/16 *Bos indicus* breed composition. Animals are enrolled in THE annually and are placed in either spring (January 1 to June 30) or fall (July 1 to December 31) calving seasons, as defined by ASA. Animals are eligible for enrollment starting at 2 yr old. Status codes provide information for each enrollment to differentiate between cows and heifers, embryo transfer donors and recipients, and to identify females not exposed or bred to be moved to another year or season. Productivity codes include information about calving, weaning, and cow disposal. Both status and productivity codes are described in detail by Giess et al. (2021). In 2009, compliance for THE was implemented so that all females had to be enrolled with a productivity or disposal code based on set deadlines for each enrollment season. To ensure consistency of calving records, only animals enrolled in 2009 and after were used in our subsequent analyses (*n* = 1,606,815). All enrollments for embryo transfer donors and recipients and all females enrolled after 2021 were removed from the dataset. Individual animal breeding records, or herd exposure records that indicate a breeding season start date or date of artificial insemination are not recorded by ASA THE. Figure 1 illustrates the number of females enrolled from the years 2012 to 2020 whose phenotypic information was included in the study. We paired THE records with performance records for all animals registered with ASA to create calving records (*n* = 2,568,200) in R using various packages in the tidyverse (Grolemund and Wickham, 2011; Wickham et al., 2019).

A subset of phenotyped cows were also genotyped on twelve different SNP arrays of varying densities but with a substantial overlapping marker set. Table 1 shows the different assays and the number of SNPs and animals genotyped by each assay. Using PLINK 1.9, we filtered genotypes to include only autosomal variants with a call rate of at least 90% on an assay-by-assay basis (Chang et al., 2015). We also removed variants with extreme Hardy–Weinburg *P*-values < 1 × 10^−50^ that were likely to be caused by genotyping errors. Genotypes were then merged based on equal positions. We then extracted the 22,179 remaining variants that were shared across all assays to be used in downstream genomic analyses. After removing individuals with > 10% missing SNPs, we retained genotypes for 33,732 phenotyped animals. Due to software constraints, our genomic analyses retained genotypes for the 25,000 animals with the greatest amount of phenotypic information. This number of genotypes should be more than sufficient for variance component estimation.

**Table 1.** Starting genotyping assays used in heritability estimation

Assay	*n* Filtered SNPs	*n* Animals
BovineHD	675,501	8
Bovine 100k A1	83,310	31,379
Bovine 50k B	40,297	25,073
Bovine 50k C	40,450	36,003
Bovine 50k R	41,732	9,419
F250	177,638	841
HDv2 B	126,653	346
HDv3 C	120,787	7,805
Simmental uLD	26,552	1,258
Simmental uLD R	22,776	14,529
SuperLDv4 C	24,524	391

This table represents the 12 manifests of genotyping arrays, their respective number of SNPs after filtering, and the number of animals genotyped on each. These arrays were then subset to a common 22,179 SNPs.

### Contemporary Groups

Animals were placed into contemporary groups to account for environmental and management differences. Groups were constructed based on the ASA owner number at the time of enrollment, enrollment year, enrollment season, and heifer status (i.e., whether this was an animal’s first opportunity to calve). We removed contemporary groups with less than five animals. This resulted in 18,383 total contemporary groups, 12,556 of which were cow groups and 5,827 were heifers. Separating cow and heifer groups helped account for management differences between groups on the same farms when calculating CDs and early calving phenotypes.

### Phenotypes

THE records and contemporary groups were used to calculate five measures of reproductive efficiency: three continuous and two discrete. For each total herd reporting entry, we attempted to associate it with a calf record from ASA’s performance data. THE dataset consisted purely of female enrollment inventories. Consequently, a cow’s CD was the day that corresponded with the calf born in the year and season of enrollment. Cows that lacked either a calf performance record or calf disposal code were assumed to be a reproductive failure. Reproductive failures remained in the herd if they were not removed from the herd’s enrollment. By restricting our dataset to post-2009, we knew that culling or removal from the herd would be accompanied with a proper disposal code. Multi-year CDs allowed us to calculate four cow fertility phenotypes and one heifer phenotype. Continuous phenotypes were CI (days between consecutive calves for each animal), first calving interval (**FCI**, CI observation between the first and second calving record for a female), and CD (day cow calved relative to the beginning of the calving season). Discrete phenotypes include discrete early calving (**DEC**, cow calved in the first 30 d of contemporary group’s calving season) and HP (cow calved as a 2-yr-old).

In addition to lifetime measurements of these phenotypes, we also analyzed CIs and CDs between a cow’s first and second calf, as this period of anestrous is the most difficult to recover from (Mercadante et al., 2003). Phenotypes were calculated in R using various packages in the tidyverse (Wickham et al., 2019). CIs were determined to be biologically impossible and were removed if they were less than 250 d (*n* = 533,155). To better estimate the start of the calving period used in CD and DEC phenotype calculations, we began counting relative to the date the third calf was born. This was aimed at removing abnormally early starts caused by premature calving events. Calves born prior to this point were considered to have a CD of zero. CDs were then broken into discrete classes to DEC phenotypes: 1) early, where calves were born in the first 30 d; 2) middle for days 31 to 60; and 3) late for days 61 to 90. Females who calved outside of a 90-d calving season were included to indicate calving outside of a 90-d season (4). Across all phenotypes, heifers were designated as females whose first enrollment year was their birth year plus two. Females who fell into this category were given a score of 1 for having a calf or 0 for not having a calf during their first calving opportunity (HP).

### Heritabilities and Genetic Correlations

We used the BLUPF90 family of programs to calculate variance components and estimate breeding values (Misztal et al., 2014). We used a mixed linear model to estimate variance components for each of these four traits. Cow traits leveraged repeated records to capture information from multiple calving seasons and estimate a permanent environmental effect. We fit the contemporary group as a single fixed effect that was defined based on a record’s herd, year, and season. Contemporary groups within operation were divided into heifer and cow groups, allowing for separate evaluation when both were included for a given phenotype. We included an age-of-dam effect for cow traits to account for conserved physiological differences between younger and older cows (BIF Guidelines, 2023). We also ran models that included binary fixed effects for the previous year dystocia and a previous year’s missed calf to test how these events altered reproductive phenotypes for a given year.

We performed univariate pedigree and single-step genomic analyses to estimate variance components for our five phenotypes of interest. The general statistical model used was as follows:


$$\begin{array}{l} y=\;X\beta +\;Za+e \end{array}$$

*y* was a vector of observations for the phenotype of interest; **X** is a design matrix relating records to their fixed effect estimates (*β*). We fit the contemporary group as the lone fixed effect in this model for heifer related traits and alongside age-of-cow effects in models of cow phenotypes. **Z** is a design matrix for random animal effects (*a*). For pedigree analyses, animal effects were distributed as *a ~ N*(0, **A**σ^2^_a_), where **A** is an additive relationship matrix. We implemented a single-step genomic analysis where animal effects were distributed as *a ~ N*(0, **H**σ^2^_a_) where **H** is a combined pedigree and genomic covariance matrix (Legarra et al., 2009). The genomic elements of this matrix leveraged the 22,179 overlapping SNPs across all genotyping arrays present in this dataset after filtering. The combined relationship matrix allowed us to leverage phenotypes from both genotyped and nongenotyped animals. For all phenotypes except HP, we fit a permanent environmental effect *p* that is distributed as *p ~ N*(0, **I**σ^2^_p_), where **I** is an identity matrix.

Additive genetic and environmental variances were estimated using GIBBSF90 + for all phenotypes. This allowed for the same statistical approach to be used for both continuous and threshold-modeled traits. For each trait, the Gibbs sampler performed 10,000 samplings, and the first 1,000 were discarded as burn-ins. We evaluated trace plots for each trait to ensure that convergence had been reached with 10,000 iterations. We estimated heritability based on the posterior estimates of genetic ($\sigma_{G}^{2}$) permanent environmental ($\sigma_{PE}^{2}$) and environmental ($\sigma_{E}^{2}$) variances as follows for traits without repeated records $h^{2}=\;\sigma_{G}^{2}\;/\;\left(\sigma_{G}^{2}+\;\sigma_{E}^{2}\right)$ and $h^{2}=\;\sigma_{G}^{2}\;/\;\left(\sigma_{G}^{2}+\sigma_{PE}^{2}+\;\sigma_{E}^{2}\right)$.

For each reproductive phenotype, we performed pairwise bivariate analysis to estimate genetic correlations. As with the univariate analyses, GIBBSF90 + was used to estimate variance and covariance components for each pair of traits. We used POSTGIBBSF90 to determine model convergence criteria and variance and covariance component estimates for all analyses.

To assess the relationship between growth traits and our reproductive traits of interest, we performed a subsequent set of bivariate analyses between each of the five reproductive phenotypes and two measures of growth: weaning weight and yearling weight. We restricted this bivariate analysis only to females with both reproductive and growth phenotypes recorded. Raw weaning and yearling weights for all animals with reproductive phenotypes were filtered to only include those within three standard deviations of the mean. We adjusted weaning weight to a standard 205-d weight and yearling weights to a 365-d weight according to BIF Guidelines. We used ASA Reporting requirements to identify acceptable weaning weights between 200 and 1,200 pounds and yearling weights between 350 and 2,000 pounds. Adjusted weights outside of this range were removed from the dataset. Models for both weights included contemporary groups that were combinations of herd, year, and season. Weaning weight models included a maternal effect and a fixed effect for age of dam class according to the BIF Guidelines. We used both GIBBSF90 + and POSTGIBBSF90 to estimate variance and covariance effects for each correlation as described above.

## Results

### Phenotypic Observations

**Figure 2.**
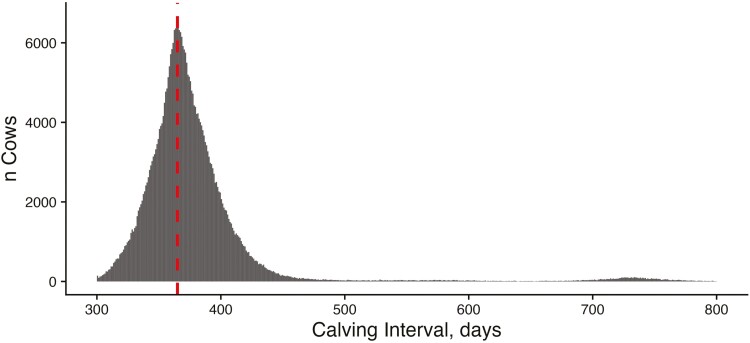
Distribution of CIs: Histogram of CI observations for females enrolled in American Simmental Association’s THE program. CIs are reported as the number of days between two consecutive calving events from the same animal. The vertical dashed line represents a 365-d interval.

**Figure 3.**
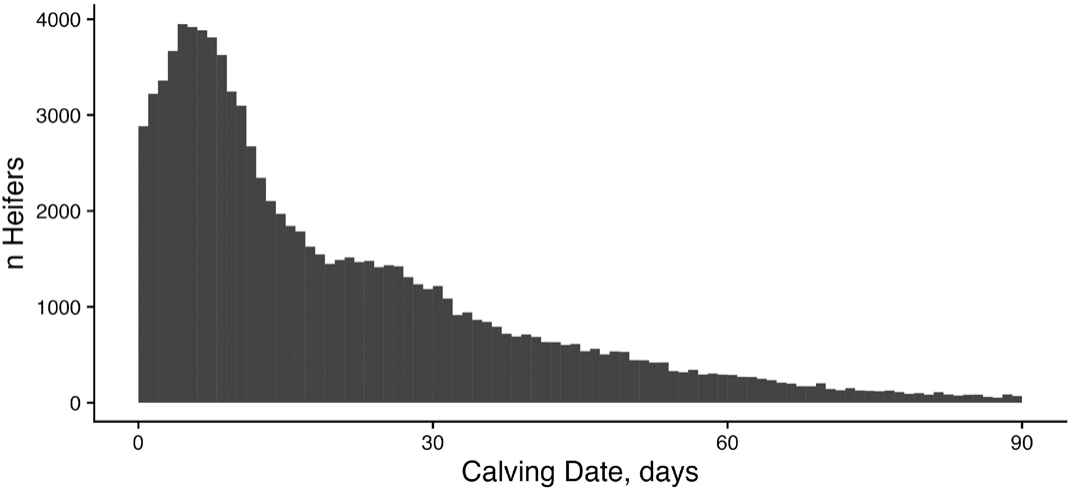
Distribution of CDs for heifers: Histogram of calving date observations for heifers enrolled in THE. CD phenotypes are the number of days from the start of the contemporary group’s calving season that an individual calved. These represent only animals in their first calving season.

**Figure 4.**
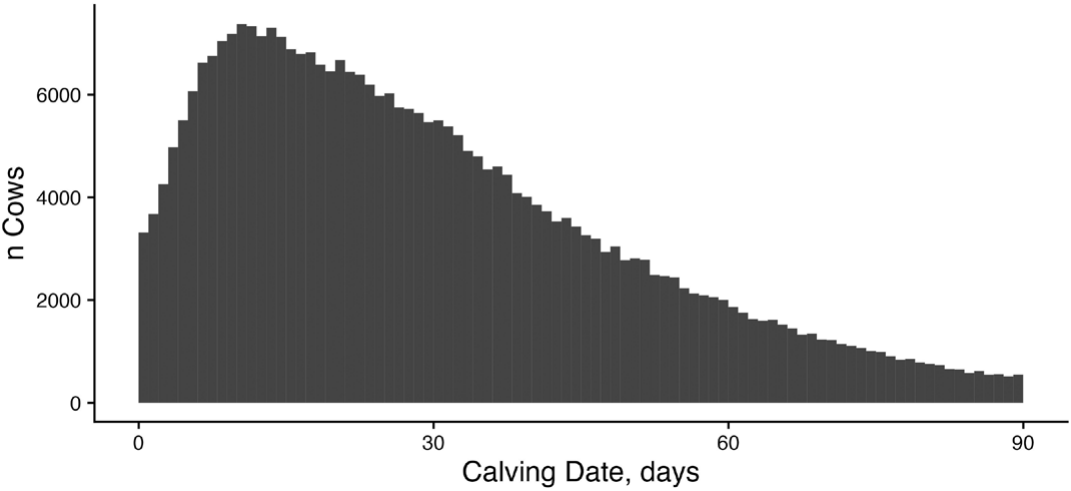
Distribution of CDs for cows: Histogram of CD observations for cows enrolled in THE. CD phenotypes are the number of days from the start of the contemporary group’s calving season that an individual calved.

We used five measures of female fertility to quantify population-level phenotypic trends in the ASA population, particularly for traits related to cow rebreeding. The mean CI for the dataset was 386 d, and the median was 370 d, with a maximum of 3,654 d. Ninety-two percent of females maintained a CI of less than 400 d (Figure 2). That said, the distribution is somewhat bimodal with a small increase in CI observations near the 2-yr, or 730-d, mark. This suggests that many females are retained in herds following a missed breeding. For both heifers and cows, the majority of females calved in the first 30 d of the contemporary group’s calving season. The mean CD for heifers was day 19 with a median of 11 d (Figure 3), while the mean for cows was day 32 with a median of 26 d (Figure 4). These discrepancies between mean and median were driven by a large number of animals with CDs of 0 or 1 based on our strategy for designating the beginning of the calving season and a handful of animals with very late CDs (>90 d). For heifers, 78.5% were classified as early calvers compared to only 57.7% of mature cows. This is likely driven by higher levels of estrus synchronization and artificial insemination in the heifer’s reproductive management compared with cows. In heifers, 16.4% and 3.8% of animals were considered middle and late calvers, respectively. This proportion of middle and late calvers was much higher in cows: 30% and 9.1%, respectively.

**Figure 5.**
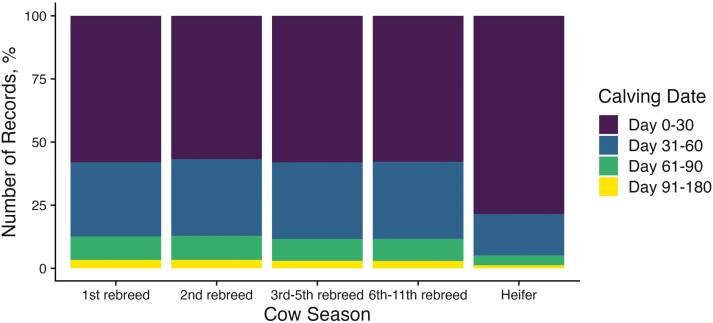
CD changes across parturitions. Stacked bar plots represent the relative proportion of animals that calved during each time period as heifers and during subsequent rebreedings. Bars are colored by discrete CD class. Third through fifth rebreedings and sixth through eleventh rebreedings are grouped together.

The remaining 1.3% of heifers and 3.2% of cows calved outside of a 90-d calving season. Despite the larger proportion of earlier calving heifers, the proportion of early, middle, and late-calving cows did not change across subsequent parturitions (Figure 5). A heifer’s first breeding season was important for success in subsequent years. We found that heifers that calved in the first 30 d of their contemporary group’s calving season went on to calve 10 d earlier, on average as mature cows (CD = 29.9 d vs. 40.2 d, *P* < 0.01).

**Figure 6.**
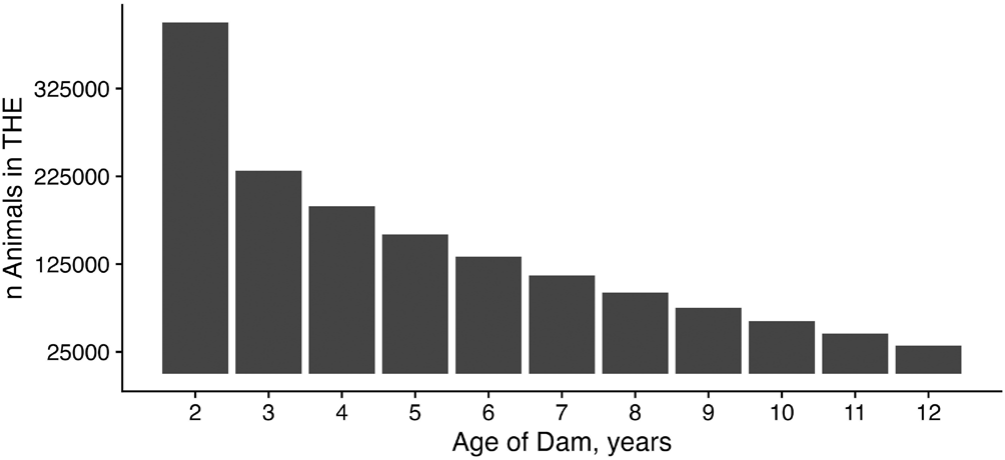
Ages of dams enrolled in THE. Number of cows enrolled in THE at different ages. We calculated a whole number age for each inventory entry across the dataset, as such cows present across multiple years would have their respective ages counted multiple times. The number of animals of each age decreases nonlinearly as a result of both attrition within herds and due to increased THE participation in recent years.

**Figure 7.**
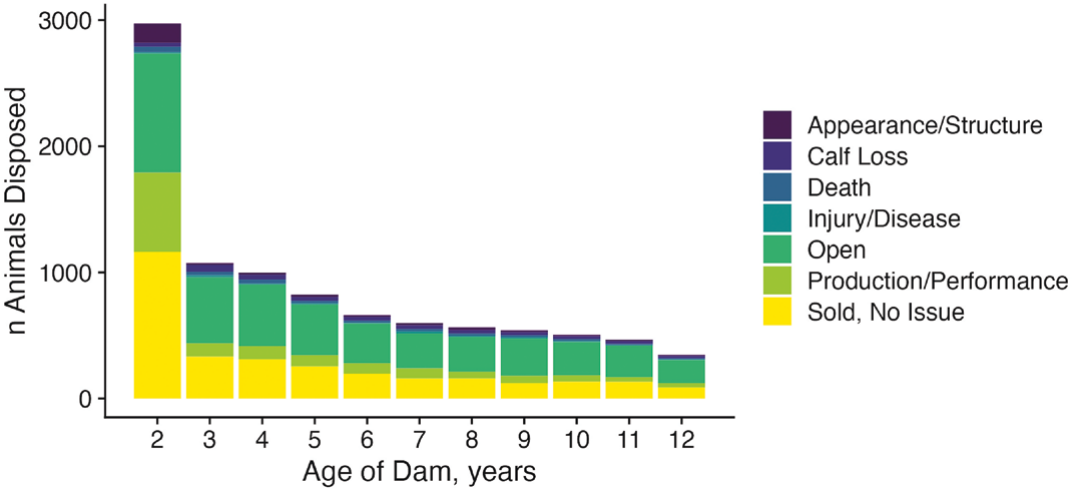
Disposal reasons by cow age. Plot of reasons for disposal for all THE females at different ages. Colors represent classes derived from the animal’s THE disposal codes.

THE data allowed us to better understand how attrition occurs in this population. As expected, we observed a steady decline in the number of animals over the course of their lifetimes. Most records in the dataset (88.9%) were from females less than 7 yr (Figure 6). Of the 75,114 enrolled animals born prior to 2015 that could have potentially reached 7 yr of age without missing a calf, only 22.4% reached their theoretical payback point. A small number of females enrolled in THE data even remained productive through age 12 (*n* = 3,806). With disposal codes being reported in THE, we found that most females are removed from the herd at 2 yr old due to either being sold for breeding purposes to other producers or because they are open (Figure 7). Based on disposal codes, failure to rebreed as the most common reason for removal from the herd, aside from transfers of an animal due to sale.

**Figure 8.**
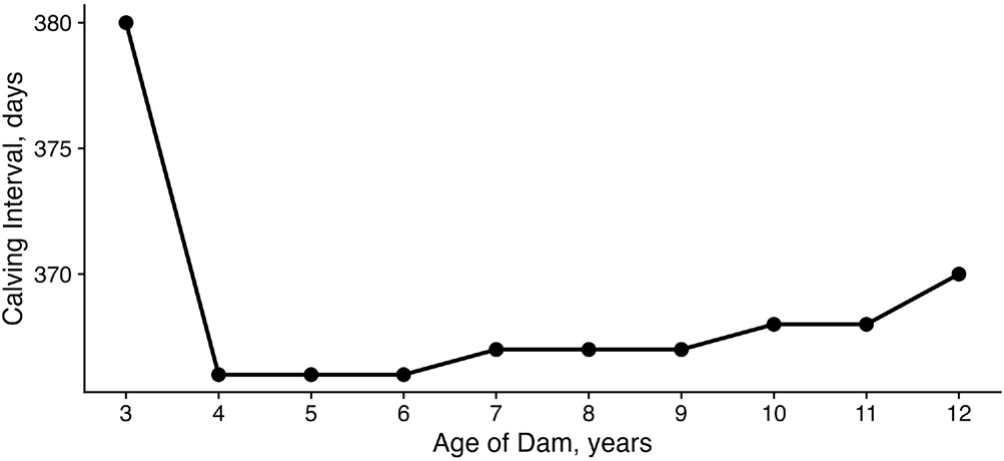
CI across productive lives. Median CI lengths for females enrolled in THE at different age points. FCI to be calculated is the time elapsed between animal’s first and second calf.

We also observed changes in females’ average CIs over their productive lives (Figure 8). CIs between ages 3 to 4 showed the largest changes in the duration of the CI, from 380 d to 368 d, respectively. This decrease is likely indicative of a common practice where CIs are indirectly elongated due to the management of heifers to calve earlier as they enter production, allowing for a longer period to recover postpartum. The next major change occurred between 11 and 12 yr of age, where CIs changed from being centered at the 365-d mark to increasing up to 376 d.

### Heritability Estimates

We estimated low-to-moderate heritabilities for each of the phenotypes of interest. Table 2 reports the full set of estimated variance components and their associated standard errors from pedigree and single-step analyses. For cows-centric phenotypes, the permanent environment effect accounted for the greatest proportion of phenotypic variance. HP was the most highly heritable reproductive phenotype, with *h*^2^ values of 0.23 and 0.24 for pedigree and genomic estimates, respectively. Due to the large size of the dataset, standard errors were quite low (0.000003 to 0.000099) for all heritability estimates. For cow-focused phenotypes, DEC (*h*^2^_pedigree_ = 0.07 and *h*^2^_genomic_ = 0.07) was the most heritable trait followed by CD (*h*^2^_pedigree_ = 0.06 and *h*^2^_genomic_ = 0.06) and CI (*h*^2^_pedigree_ = 0.04 and *h*^2^_genomic_ = 0.04). The FCI had the same genomic *h*^2^ estimate as CI with a value of 0.04 but had a slightly higher pedigree *h*^2^ estimate of 0.05. We did not observe any changes to heritability estimates when we included fixed effects for dystocia or a missed calf in the previous year.

**Table 2.** Variance components for fertility traits in ASA THE dataset

Phenotype	Method	*V*_*g*_	*V*_*p*_	*V*_*e*_	*h* ^2^
Calving date	Pedigree	26.893 ± 5.0106	73.607 ± 4.0359	382.51 ± 0.84391	0.06 ± 0.010079
	Genomic	31.451 ± 4.6590	71.169 ± 3.4326	382.36 ± 0.76896	0.06 ± 0.009265
Discrete early calving	Pedigree	0.079 ± 0.014324	0.191 ± 0.034839	0.924 ± 0.23268	0.07 ± 0.014758
	Genomic	0.078 ± 0.015203	0.191 ± 0.034852	0.917 ± 0.230760	0.07 ± 0.015451
Calving interval	Pedigree	550.74 ± 52.637	270.51 ± 65.176	12,880.0 ± 71.832	0.04 ± 0.003699
	Genomic	540.22 ± 56.468	296.50 ± 63.292	12,881.0 ± 73.688	0.04 ± 0.003954
First calving interval	Pedigree	40.226 ± 11.598	91.857 ± 9.972	758.38 ± 8.3274	0.05 ± 0.013841
	Genomic	34.737 ± 7.980	102.55 ± 10.173	753.18 ± 8.390	0.04 ± 0.009604
Heifer pregnancy	Pedigree	0.296 ± 0.077072	NA	1.011 ± 0.004975	0.22 ± 0.039278
	Genomic	0.324 ± 0.074052	NA	1.011 ± 0.004953	0.24 ± 0.037215

Genetic variance (*V*_*g*_), environmental variance (*V*_*p*_), error variance (*V*_*e*_), and heritability (*h*^2^) estimates with posterior standard deviations for all phenotypes.

### Phenotypic and Genetic Correlations

We observed both positive and negative genetic correlations between the phenotypes in bivariate analyses (Table 3). Binary HP phenotypes were minimally phenotypically correlated with all other cow-focused phenotypes (r_P_ = 0.004 to 0.03). A moderate genetic correlation (r_G_ = 0.19) existed between HP and CD, indicating that shared genetics impact successful breeding as a heifer and lifelong early calving. CI and HP were negatively genetically correlated (r_G_ = −0.07) but showed minimal phenotypic correlation (r_P_ = 0.004). In all other cases, the directionality of phenotypic correlations matched those of the genetic correlations, varying in magnitudes. As expected, the strongest genetic correlation existed between CD and DEC (r_G_ = 0.96) due to the direct derivative relationship between the two phenotypes. CI and FCI showed low phenotypic correlations (r_P_ = 0.13), but moderately high genetic correlations (r_G_ = 0.47). The next highest genetic correlation existed between FCI and CD (r_G_ = 0.34), suggesting that a genetic predisposition for rebreeding after a cow’s first calf is shared with early calving throughout its productive lifetime. Estimates of genetic correlations all had very small associated standard errors (0.000011 to 0.000273). A cow’s FCI showed weak phenotypic correlations with both CD and CI (0.07 and 0.13, respectively). CD and DEC were similarly correlated to HP (r_G_ = 0.07 to 0.19).

**Table 3.** Heritabilities, genetic, and phenotypic correlations

Phenotype	Heifer pregnancy	Calving date	Discrete early calving	First calving interval	Calving interval
Heifer pregnancy	**0.24** ± 0.000094	0.19 ± 0.000200	0.07 ± 0.000124	0.01 ± 0.000011	−0.07 ± 0.000273
Calving date	0.03	**0.06** ± 0.000003	0.96 ± 0.000090	0.34 ± 0.000075	0.05 ± 0.000151
Discrete early calving	0.03	0.93	**0.07** ± 0.000017	0.17 ± 0.000029	0.10 ± 0.000149
First calving interval	0.02	0.07	0.07	**0.04** ± 0.000029	0.47 ± 0.000231
Calving interval	0.004	0.15	0.15	0.13	**0.04 ± **0.000005

Heritabilities and their associated standard errors are reported on the table’s diagonal and are bolded. Upper off-diagonal values are genetic correlations and associated standard errors. Lower off-diagonal elements are phenotypic correlations.

We also calculated correlations between the five reproductive phenotypes and two measures of growth, weaning weight and yearling weight. These are reported in Table 4. We found minimal correlation between weaning weight and our measurements of reproductive performance (r_G_ = −0.023 to 0.007). Correlations between yearling weight and our reproductive traits were much more variable and represented much larger magnitudes than did weaning weight (r_G_ = −0.091 to 0.235). HP showed the highest genetic correlation with yearling weight (r_G_ = 0.235). The FCI also showed a modest relationship with yearling weight (r_G_ = −0.091), indicating that more mature and heavier heifers at 1 yr of age were more likely to rebreed earlier after having their first calf. The magnitudes of these relationships tailed off for other reproductive phenotypes.

**Table 4.** Correlations between reproductive phenotypes and growth traits

Phenotype	Weaning weight	Yearling weight
Heifer pregnancy	−0.023 ± 0.008491	0.235 ± 0.08758
Calving date	−0.013 ± 0.005642	0.066 ± 0.057977
Discrete early calving	−0.012 ± 0.0056099	0.076 ± 0.041372
First calving interval	−0.004 ± 0.004054	−0.091 ± 0.040377
Calving interval	0.007 ± 0.005642	0.021 ± 0.10037

Genetic correlation values with posterior standard deviations.

## Discussion

Our observations of phenotypic distributions were largely in line with expectations. For example, the median CI of 370 d indicated that the vast majority of females present in the dataset were successfully producing one calf per year throughout their time in enrollment. This indicates that seedstock producers are limiting the number of open females in their cow herds and only retaining productive females each year.

We observed low-to-moderate heritability estimates for all studied phenotypes, in line with estimates across different populations and fertility-related phenotypes. CD was one of the least heritable traits in our population. Previous studies have reported a broad range of heritability estimates for this trait. However, most of them tend to be low. Morris et al. (2000) calculated heritability to be 0.091 ± 0.036 in a population of Angus cattle, while Buddenberg et al. (1990) estimated a wide heritability range from 0.13 to 0.39 in a population of Hereford cattle. This low heritability CD makes phenotypic selection nearly impossible despite the clear economic relevance of calving early in the season. This economic signal is driven by the fact that calves born early in the season are typically heavier at weaning (Cushman et al., 2013). DEC was also lowly heritable in this population, which was expected based on its direct relationship to CD. Interestingly, we found that DEC was slightly more heritable than CD.

HP was the most heritable trait that we observed, falling into the moderately heritable range with *h*^2^ values of 0.23 and 0.24 for pedigree and genomic information, respectively. In two previous studies of Angus cattle, heritabilities were estimated to be 0.27 and 0.21 (Doyle et al., 2000). However, other studies have estimated heritability to be more variable, ranging from 0.12 to 0.53 across different populations (Van Melis et al., 2010; McAllister et al., 2011; Boldt et al., 2018). HP has typically been modeled as a binary observation (i.e., Did a female calve as a 2-yr-old?). Other work has used continuous phenotypes such as age at first calving or age at puberty as indicator. Heritability estimates for age at first calving and age at puberty are similar to HP heritability estimates from our study, with estimates ranging from 0.08 to 0.24 for age at first calving and 0.07 to 0.67 for at age puberty (Martin et al., 1992; Gutiérrez et al., 2002; Martínez-Velázquez et al., 2003).

We found both positive and negative phenotypic correlations among the studied traits (r_G_ from 0.004 to 0.96). This wide range in correlations indicates that while these phenotypes are all associated with cow fertility, they may be controlled by largely different genetic architectures. CI and CD were the least correlated cow-focused phenotypes with a value of 0.05. The key difference between these two phenotypes is that a favorable CD can be the result of a reproductive failure, and a female is shifted to calve in a different season or year (i.e., CD could be zero but CI could be 730). CI can capture this information because this phenotype will be elongated (>365 d), and this record will have a negative impact on the female’s projected productivity. CI and HP were lowly genetically correlated, but it is worth mentioning an HP phenotype does not have a matched CI observation. Because of how heifers are managed to calve earlier in the season, indirectly, CI is impacted by heifer calving records since most occur early in the calving season. This management practice, although beneficial for the assurance of a successful rebreeding, can indirectly elongate CI phenotypes since heifers are managed to calve earlier than mature cows but then bred back for the next season at the same time as the mature cows. Interestingly, an animal’s genetic potential for CD was more positively correlated with its FCI (0.34) than with CI throughout the entirety of an animal’s lifetime (0.05). We also found that heifers that conceived early in their first breeding season went on to have shorter CDs throughout the course of their lives.

Genetic correlations between growth traits and fertility were variable. Correlations with weaning weight were all effectively zero, indicating that direct calf growth is not likely to be affected by selection for these traits. Interestingly, the magnitude of genetic correlations was larger for yearling weight. HP and yearling weight showed the strongest correlation (r_G_ = 0.235). This is likely driven by the relationship between yearling weight and puberty attainment, as heifers that are heavier at 365 d of age are likely to be more mature and primed for breeding in the following months. These differences also appeared to persist to the animal’s first rebreeding, where yearling weight was negatively correlated with an animal’s FCI (r_G_ = −0.091). However, the remaining correlations between the two growth traits and the five reproductive phenotypes were centered around zero, indicating that selection for growth may not impact sustained cow fertility and productivity.

While THE is a valuable tool for collecting reproductive phenotypes in cattle, a lack of detail in the data collected presents limitations for genetic evaluation. The collection of reproductive phenotypes in beef cattle populations did not begin in earnest until the implementation of inventory-based reporting. Even with the implementation of WHR, the beef industry lags behind the dairy industry in collecting more granular breeding records, specifically those related to actual breeding dates or service types (AI vs. natural service) that would allow for more precise measurements of these phenotypes. At the most basic level, collecting a breeding date, or even the start of the breeding season for each THE entry would be useful in calculating more precise and accurate phenotypes related to CD. This would also allow for the joint analysis of fertility phenotypes with gestation lengths. Collecting palpation records or approximate fetal age could aid in estimating gestation lengths when breeding dates are not reported. This information would allow for the back calculation of breeding dates to increase precision in developing contemporary groups when accounting for management differences. Further, if detailed breeding records were collected, CD could account for potential differences in conception from natural service versus AI versus estrus synchronization. We expect that with the ability to differentiate animals with breeding records would show which animals respond best to each type of service and change the distribution of CD. CD distributions in natural service settings would likely appear the most similar to the distributions observed in this work. We would expect that estrous synchronization protocols would shift CD closer to day 0 and increase the number of early calving records. As beef cattle breed associations look for ways to improve data collection, the inclusion of breeding records, including estrous synchronization, artificial insemination, or natural service, and dates for inventory-based reporting would allow for a more detailed investigation of the cow productivity phenotypes.

## Conclusions

We explored five phenotypes calculated from the American Simmental Association’s Total Herd Reporting dataset that are indicators of cow fertility. The unbiased reporting required in THE allowed us to understand culling decisions and cow attrition at the population level. Most seedstock Simmental producers maintain close to a 365-d CI on average, but room for improvement still exists. CD for heifers was predictive of future reproductive performance. Each of these phenotypes was lowly to moderately heritable. All phenotypes showed low-to-moderate phenotypic and genetic correlations with one another. This suggests that genetic improvement is possible for these traits if breed associations develop genetic evaluations and genetic selection tools for them. To maximize the effectiveness of these tools, breed associations should consider strategies for collecting more granular breeding records. Future work will need to identify optimal modeling strategies for genetic predictions and potential inclusion into economic selection indexes.

## Abbreviations

ASA: American Simmental Association
CD: calving date
CI: calving interval
DEC: discrete early calving
FCI: first calving interval
GWAS: genome-wide association study
HP: heifer pregnancy
THE: total herd enrollment

## References

[CIT0001] Berry, D. P., E. Wall, and J. E. Pryce. 2014. Genetics and genomics of reproductive performance in dairy and beef cattle. Animal. 8:105–121. doi: https://doi.org/10.1017/S1751731114000743 24703258

[CIT0002] BIF Guidelines. 2023. Guidelines for uniform beef improvement programs. BIF Guidelines Wiki. June 6, 2023. Manhattan, Kansas. http://guidelines.beefimprovement.org/index.php?title=Guidelines_for_Uniform_Beef_Improvement_Programs&oldid=2679

[CIT0003] Boldt, R. J., S. E. Speidel, M. G. Thomas, and R. M. Enns. 2018. Genetic parameters for fertility and production traits in Red Angus cattle. J. Anim. Sci. 96:4100–4111. doi: https://doi.org/10.1093/jas/sky294 30204881 PMC6162596

[CIT0004] Bourdon, R. M., and J. S. Brinks. 1982. Genetic, environmental and phenotypic relationships among gestation length, birth weight, growth traits and age at first calving in beef cattle. J. Anim. Sci. 55:543–553. doi: https://doi.org/10.2527/jas1982.553543x 7130063

[CIT0005] Boyer, C. N., A. P. Griffith, K. L. DeLong, C. N. Boyer, A. P. Griffith, and K. L. DeLong. 2020. Reproductive failure and long-term profitability of spring- and fall-calving beef cows. doi: https://doi.org/10.22004/AG.ECON.298435

[CIT0006] Buddenberg, B. J., C. J. Brown, and A. H. Brown. 1990. Heritability estimates of calving date in Hereford cattle maintained on range under natural mating. J. Anim. Sci. 68:70–74. doi: https://doi.org/10.2527/1990.68170x 2303402

[CIT0007] Cammack, K. M., M. G. Thomas, and R. M. Enns. 2009. Reproductive traits and their heritabilities in beef cattle. Prof. Anim. Scientist. 25:517–528. doi: https://doi.org/10.15232/s1080-7446(15)30753-1

[CIT0008] Chang, C. C., C. C. Chow, L. C. Tellier, S. Vattikuti, S. M. Purcell, and J. J. Lee. 2015. Second-generation PLINK: rising to the challenge of larger and richer datasets. GigaScience 4:7. doi: https://doi.org/10.1186/s13742-015-0047-8 25722852 PMC4342193

[CIT0009] Cundiff, L. V., M. D. MacNeil, K. E. Gregory, and R. M. Koch. 1986. Between- and within-breed genetic analysis of calving traits and survival to weaning in beef cattle. J. Anim. Sci. 63:27–33. doi: https://doi.org/10.2527/jas1986.63127x 3733574

[CIT0010] Cushman, R. A., L. K. Kill, R. N. Funston, E. M. Mousel, and G. A. Perry. 2013. Heifer calving date positively influences calf weaning weights through six parturitions. *J. Anim. Sci.* 91:4486–4491. doi: https://doi.org/10.2527/jas.2013-6465 23825337

[CIT0011] Diskin, M. G., and D. A. Kenny. 2014. Optimising reproductive performance of beef cows and replacement heifers. Animal. 8:27–39. doi: https://doi.org/10.1017/S175173111400086X 24703426

[CIT0012] Doyle, S. P., B. L. Golden, R. D. Green, and J. S. Brinks. 2000. Additive genetic parameter estimates for heifer pregnancy and subsequent reproduction in Angus females. J. Anim. Sci. 78:2091–2098. doi: https://doi.org/10.2527/2000.7882091x 10947093

[CIT0013] Garcia, J., D. Anderson, A. Herring, and D. Riley. 2015. Economic analysis of selecting for cow longevity. doi:10.22004/ag.econ.14695

[CIT0014] Giess, L. K., M. G. Thomas, S. E. Speidel, M. M. Culbertson, W. R. Shafer, S. C. McGuire, and R. M. Enns. 2021. Whole herd reporting data from the American simmental association as a data source for heifer pregnancy phenotypes. Transl. Anim. Sci. 5:S199–S203. doi: https://doi.org/10.1093/tas/txab152

[CIT0015] Grolemund, G., and H. Wickham. 2011. Dates and times made easy with lubridate. J. Stat. Softw. 40:1–25. doi: https://doi.org/10.18637/jss.v040.i03

[CIT0016] Gutiérrez, J. P., I. Alvarez, I. Fernández, L. J. Royo, J. Díez, and F. Goyache. 2002. Genetic relationships between calving date, calving interval, age at first calving and type traits in beef cattle. Livest. Prod. Sci. 78:215–222. doi: https://doi.org/10.1016/s0301-6226(02)00100-8

[CIT0017] Legarra, A., I. Aguilar, and I. Misztal. 2009. A relationship matrix including full pedigree and genomic information. J. Dairy Sci. 92:4656–4663. doi: https://doi.org/10.3168/jds.2009-2061 19700729

[CIT0018] Lesmeister, J. L., P. J. Burfening, and R. L. Blackwell. 1973. Date of first calving in beef cows and subsequent calf production. J. Anim. Sci. 36:1–6. doi: https://doi.org/10.2527/jas1973.3611

[CIT0019] MacNeil, M. D., T. W. Geary, G. A. Perry, A. J. Roberts, and L. J. Alexander. 2006. Genetic partitioning of variation in ovulatory follicle size and probability of pregnancy in beef cattle. J. Anim. Sci. 84:1646–1650. doi: https://doi.org/10.2527/jas.2005-698 16775047

[CIT0020] Martin, L. C., J. S. Brinks, R. M. Bourdon, and L. V. Cundiff. 1992. Genetic effects on beef heifer puberty and subsequent reproduction. J. Anim. Sci. 70:4006–4017. doi: https://doi.org/10.2527/1992.70124006x 1474037

[CIT0021] Martínez-Velázquez, G., K. E. Gregory, G. L. Bennett, and L. D. Van Vleck. 2003. Genetic relationships between scrotal circumference and female reproductive traits. J. Anim. Sci. 81:395–401. doi: https://doi.org/10.2527/2003.812395x 12643482

[CIT0022] Mathews, K. H. Jr, and S. D. Short. 2001. The beef cow replacement decision. J. Agribus. 19:191–211. doi: https://doi.org/10.22004/ag.econ.14695

[CIT0023] McAllister, C. M., S. E. Speidel, D. H. Crews, Jr, and R. M. Enns. 2011. Genetic parameters for intramuscular fat percentage, marbling score, scrotal circumference, and heifer pregnancy in Red Angus cattle. J. Anim. Sci. 89:2068–2072. doi: https://doi.org/10.2527/jas.2010-3538 21278121

[CIT0024] Mercadante, M. E. Z., I. U. Packer, A. G. Razook, J. N. S. G. Cyrillo, and L. A. Figueiredo. 2003. Direct and correlated responses to selection for yearling weight on reproductive performance of Nelore cows. J. Anim. Sci. 81:376–384. doi: https://doi.org/10.2527/2003.812376x 12643480

[CIT0025] Minick Bormann, J., and D. E. Wilson. 2010. Calving day and age at first calving in Angus heifers. J. Anim. Sci. 88:1947–1956. doi: https://doi.org/10.2527/jas.2009-2249 20154171

[CIT0026] Misztal, I., S. Tsuruta, D. Lourenco, and Y. Masuda. 2014. BLUPF90 family of programs. http://nce.ads.uga.edu/wiki/lib/exe/fetch.php?media=blupf90_all8.pdf

[CIT0027] Morris, C. A., J. A. Wilson, G. L. Bennett, N. G. Cullen, S. M. Hickey, and J. C. Hunter. 2000. Genetic parameters for growth, puberty, and beef cow reproductive traits in a puberty selection experiment. N. Z. J. Agric. Res. 43:83–91. doi: https://doi.org/10.1080/00288233.2000.9513411

[CIT0029] Rowan, T. N. 2022. Invited review: genetic decision tools for increasing cow efficiency and sustainability in forage-based beef systems. Appl. Anim. Sci. 38:660–670. doi: https://doi.org/10.15232/aas.2022-02306

[CIT0030] Snelling, W. M., B. L. Golden, and R. M. Bourdon. 1995. Within-herd genetic analyses of stayability of beef females. J. Anim. Sci. 73:993–1001. doi: https://doi.org/10.2527/1995.734993x 7628978

[CIT0031] Van Melis, M. H., J. P. Eler, G. J. M. Rosa, J. B. S. Ferraz, L. G. G. Figueiredo, E. C. Mattos, and H. N. Oliveira. 2010. Additive genetic relationships between scrotal circumference, heifer pregnancy, and stayability in Nellore cattle. J. Anim. Sci. 88:3809–3813. doi: https://doi.org/10.2527/jas.2009-2127 20656970

[CIT0032] Wickham, H., M. Averick, J. Bryan, W. Chang, L. McGowan, R. François, G. Grolemund, A. Hayes, L. Henry, J. Hester, et al. 2019. Welcome to the tidyverse. J. Open Source Softw. 4:1686. doi: https://doi.org/10.21105/joss.01686

